# Annotations

**Published:** 1889-06-22

**Authors:** 


### Annotations.

**The "Damien" Memorial.** That some memorial should be instituted to perpetuate, and make an object lesson for future generations, of the noble deed of Father Damien, every reasonable human being will affirm with heart and mind. Nothing need be said of this unknown priest, except that he resolved upon and carried out a way of life which placed him then and for ever among the greatest and noblest the human race has ever seen. Father Damien was no writer of prose or verse who amused the leisure of the toilers, or drove away the *ennui* of the idlers with his wit. He was no soldier who filled the world with the sound of his deeds, and weaker men than himself with an unworthy dread of his power. He was a man, like many others whom fame never overtakes, who gave his life to serve unhappy people because they lacked service and could not pay for it, instead of seeking to push his own fortunes in the way that was open to him. No brave Roman holding the bridge against all comers had a more heroic heart than he. The Prince of Wales has done well to give the weight of his influence to the object of raising a worthy memorial to such a man. The proposal to found a Leper Ward in connection with one of the London hospitals demands further consideration, but such an establishment in our Indian Empire, where hundreds of lepers are dragging out a miserable existence, would be welcomed by all who deplore their unhappy and hopeless fate. It seems passing strange that such large numbers of English physicians and surgeons as have spent the best part of their lives in that country where lepers so abound should have done so little to make clear the nature of leprosy, and to bring it within range of cure. But it is not so strange as it seems. Disease in its protean forms is profoundly mysterious, and requires for its philosophic comprehension a rare combination of mental and moral qualities. To observe the phenomena of morbid processes, and to carry out recognised and routine treatment is one thing; but to trace every morbid process to its true source, to discover the relationship between different conditions, to clearly comprehend and define not only the progress and the conclusion, but also the nature and origin of any given disease, to put it into its class with scientific accuracy and precision, and then to treat it by such methods as experience has shown to be rational, because generally successful, is quite another. It requires, in fact, what in literature and other spheres men call "genius." "Genius is an infinite capacity for taking pains," no doubt, but it is something more. To be a scientific and philosophic physician, a man must not only have the "infinite capacity for taking pains," but he must have the something more which constitutes true genius. When it is remembered how seldom among the thousands of literary men it can be said with truth "here is a man of genius," how few works of literature bear the stamp of immortality, we need not wonder that medicine, which is almost the newest of the scientific arts, should not produce men of genius in every country town. There may not be in India one single man among all the distinguished medical practitioners of that country who has the natural endowments and the professional attainments which fit him for the study of so peculiar and grievous a disease as leprosy. In London there is at the present moment one man whom the whole medical profession would with united voice name as the man of all others who has the natural capacity of mind, the trained observing skill, the breadth of professional knowledge, and the infinite capacity for taking pains, which ought to be demanded of him who is entrusted by civilisation itself with the high and great duty of investigating, and, if possible, treating successfully, this world-old and classical, but dreadful disease. That man, we need not say, is Mr. Jonathan Hutchinson. Mr. Hutchinson was at the Mansion House meeting, and it is much to be desired for the sake of the work which is to be Father Damien's memorial that Mr. Hutchinson may be prevailed upon to undertake the conduct of the investigations which it is so desirable to commence at once.

**The Proposed Pasteur Institute.** There is a strong feeling on the part of a section of the medical profession that an institute for carrying out the treatment of M. Pasteur for hydrophobia ought to be established in this country. If that proposal involved neither pain to animals nor danger to human beings there would be little reason to object to it; nay, it might even be decidedly encouraged as tending to the enlargement of the field of experimental therapeutics. But there is one Pasteur Institute already in full work; and as that is the occasion of incalculable suffering and misery to many animals, as, moreover, its treatment is fraught with much risk to human beings, and as, still more, there is no really convincing proof that the mortality from hydrophobia is in any degree lessened thereby, it is expedient that, for the present at least, the one Pasteur Institute shall suffice. We have more than once pointed out in these pages the antecedent improbability of Pasteur's method proving really an antiphlogistic to rabies. If in spite of such antecedent improbability experiment should nevertheless conclusively demonstrate that the practice of Pasteur is really a life-saving proceeding, we shall bow to undoubted fact. In the meantime, however, it is worth consideration what a very peculiar series of circumstances we are asked to put our trust in. Rabies, like scarlet fever or smallpox, is admittedly a bacterial disease. Every medical man will grant that, other things being equal, scarlet fever or smallpox will develop in a given individual with greater or less promptitude and energy in proportion to the quantity and activity of the bacterial virus received by the subject of infection. In other words, a large quantity of a very energetic virus will develop more quickly and energetically, under like conditions, than a smaller quantity of a decidedly attenuated virus. This is granted by all medical men, including even homœopaths, who might, on Hahnemann's principles, offer a plausible objection to it. But what does M. Pasteur ask us to believe? That in the case of rabies the bacterial virus will act quite differently, nay, even antipodally, from what it does in every other known bacterial disease. The virus from the dog's bite is admittedly a highly active virus; it is introduced into the system one, two, or several days in advance of the antidote. The Pasteur inoculating fluid is a highly attenuated virus; it is introduced into the circulation one, two, or several days later than the stronger virus. Both the viruses seek the same pabulum in the system. The one that reaches it first consumes it more or less completely, and the other coming up afterwards, finds nothing to feed upon and dies. We are asked to believe by Pasteur and his disciples that the weak virus, starting a day or two, or several days, later in the race will outrun the stronger one, and seizing first upon the pabulum, for which the stronger is still more fiercely hungering, will destroy it before the latter can come up. That is to say, a full-grown and ravenously hungry wolf, starting at dusk for a sheepfold five miles off, will be outrun by a mere cub which is not very hungry, though the latter shall not start until midnight. We will believe this when M. Pasteur has proved it; and we will believe that the earth is a plane when Mr. John Hampden, the ingenious advocate of the plane theory, has done the same. There is no doubt that an effective and universal system of muzzling dogs would extirpate rabies completely. What is to prevent such a system being carried out at once?

